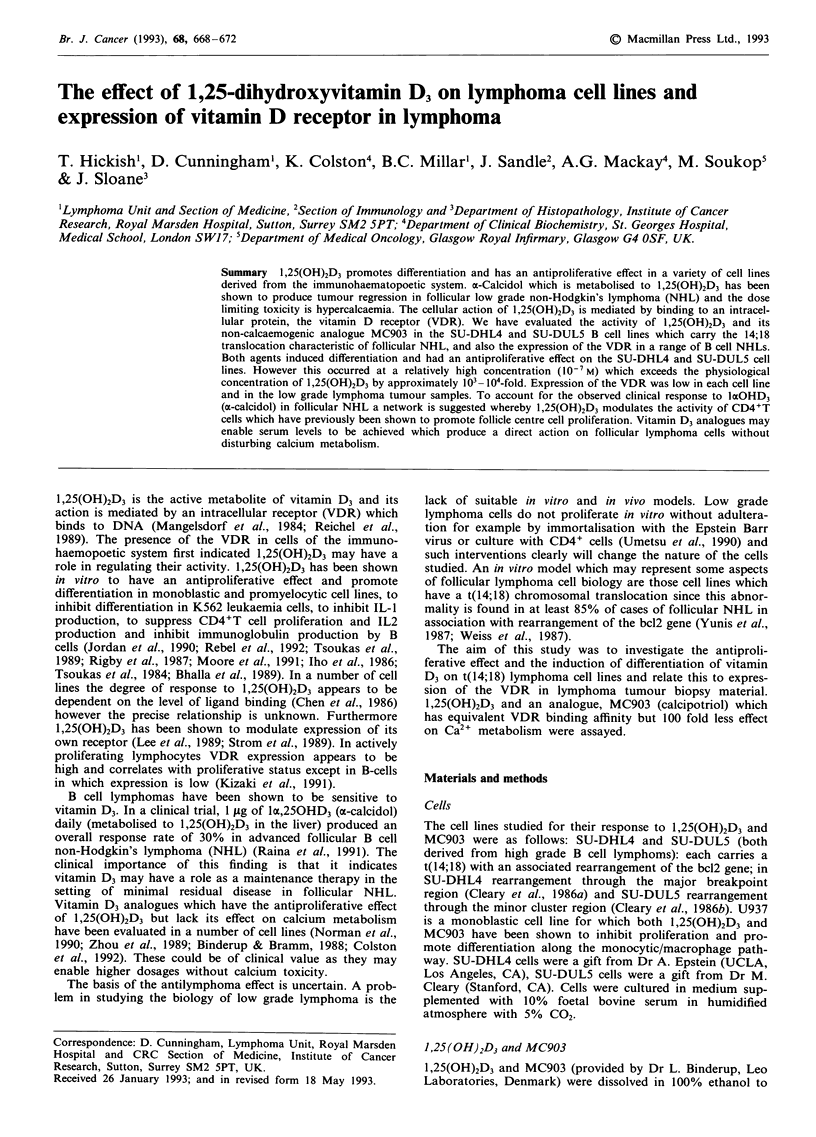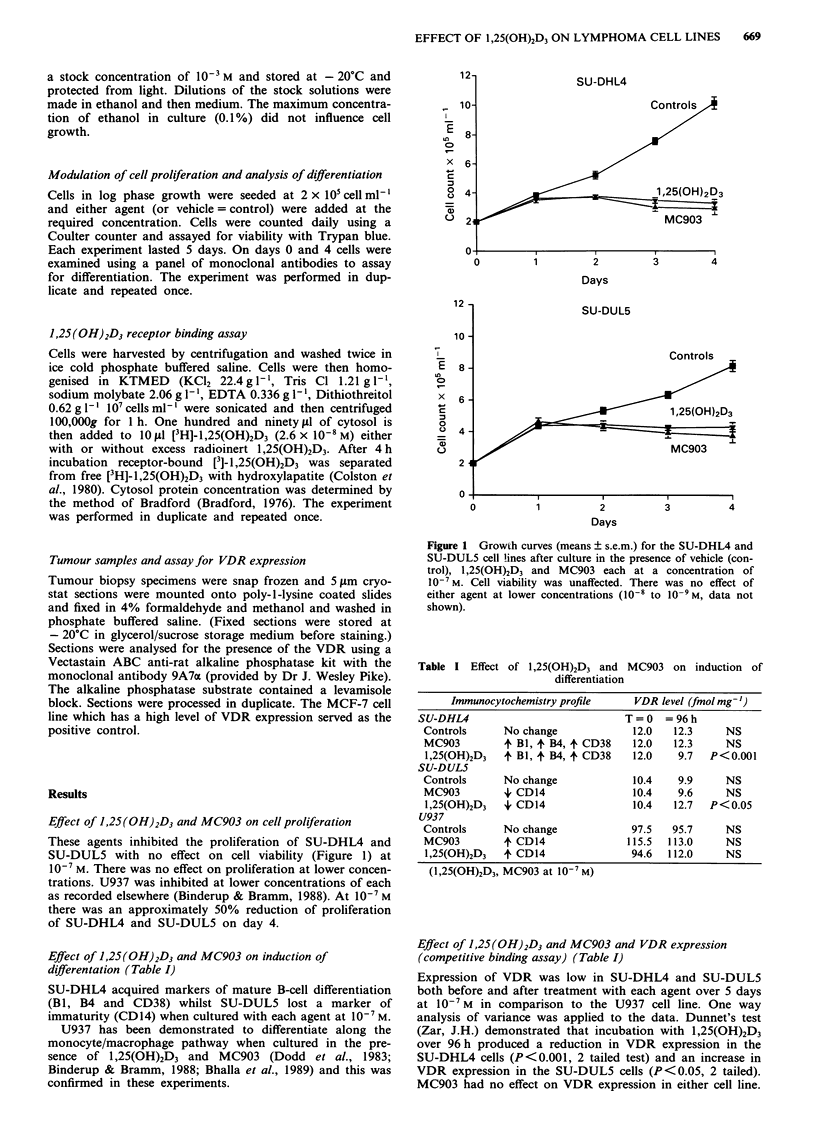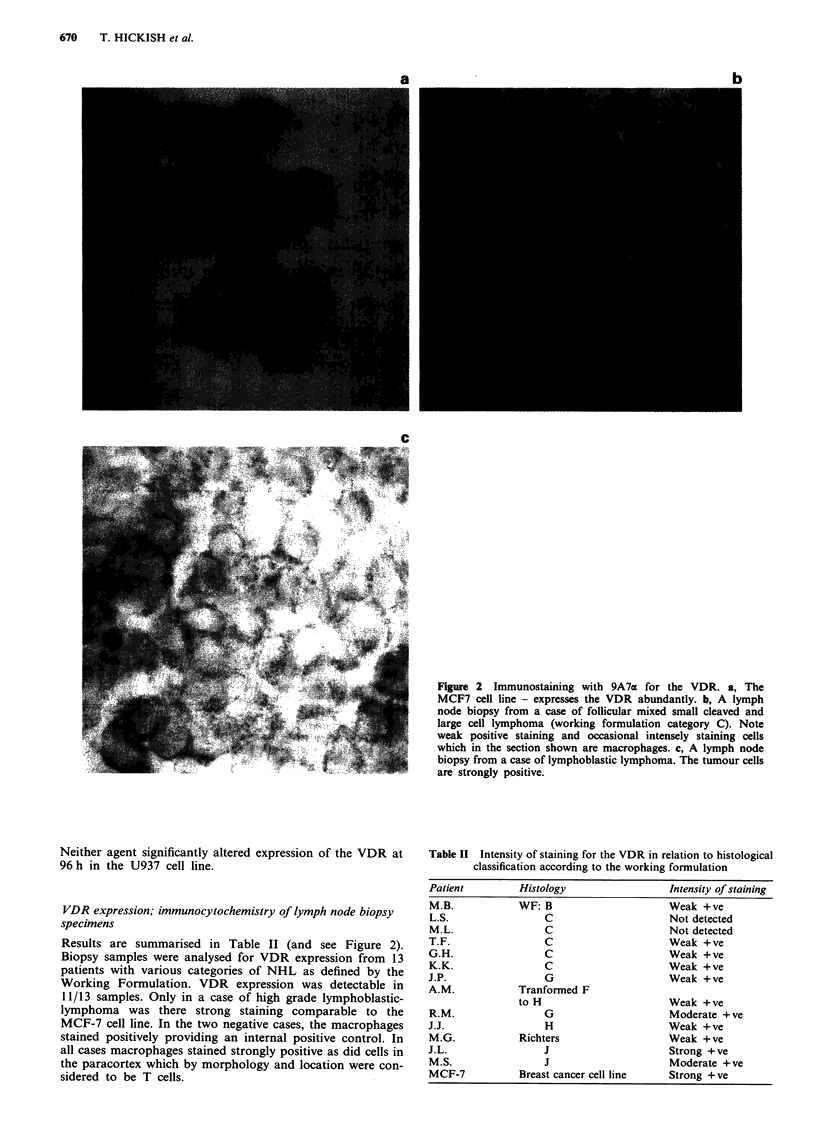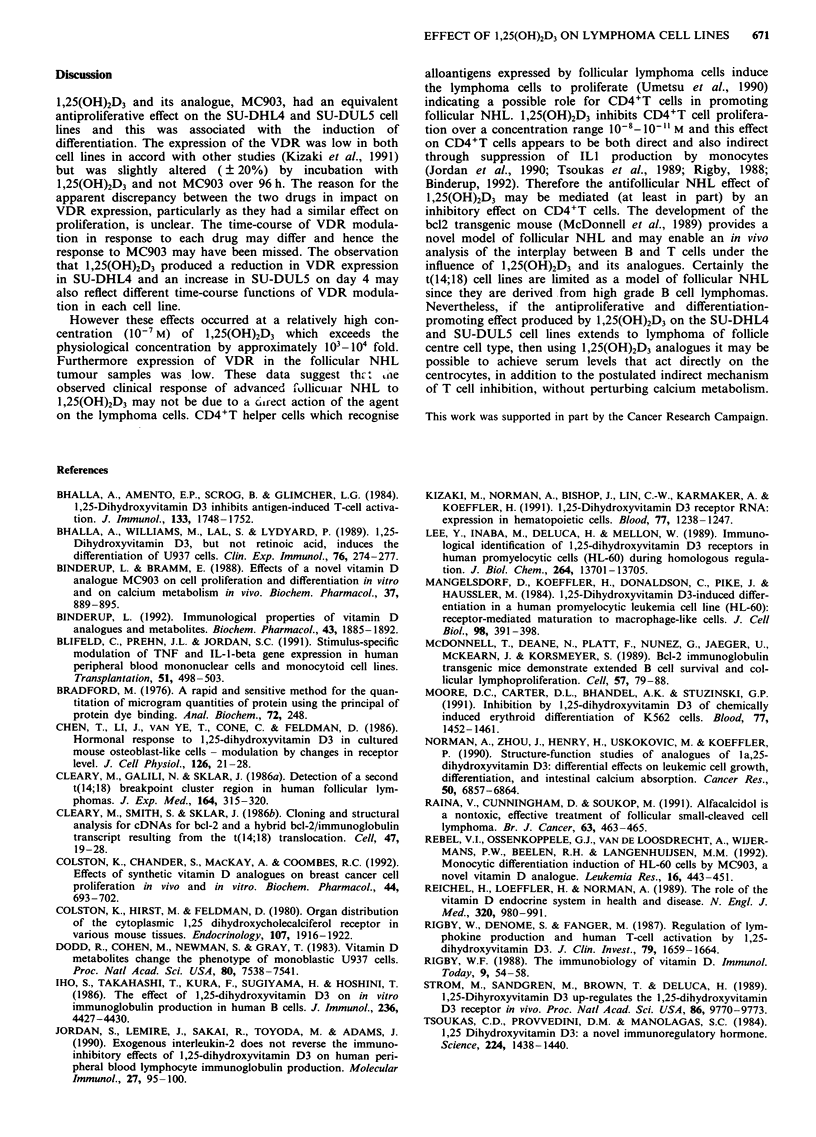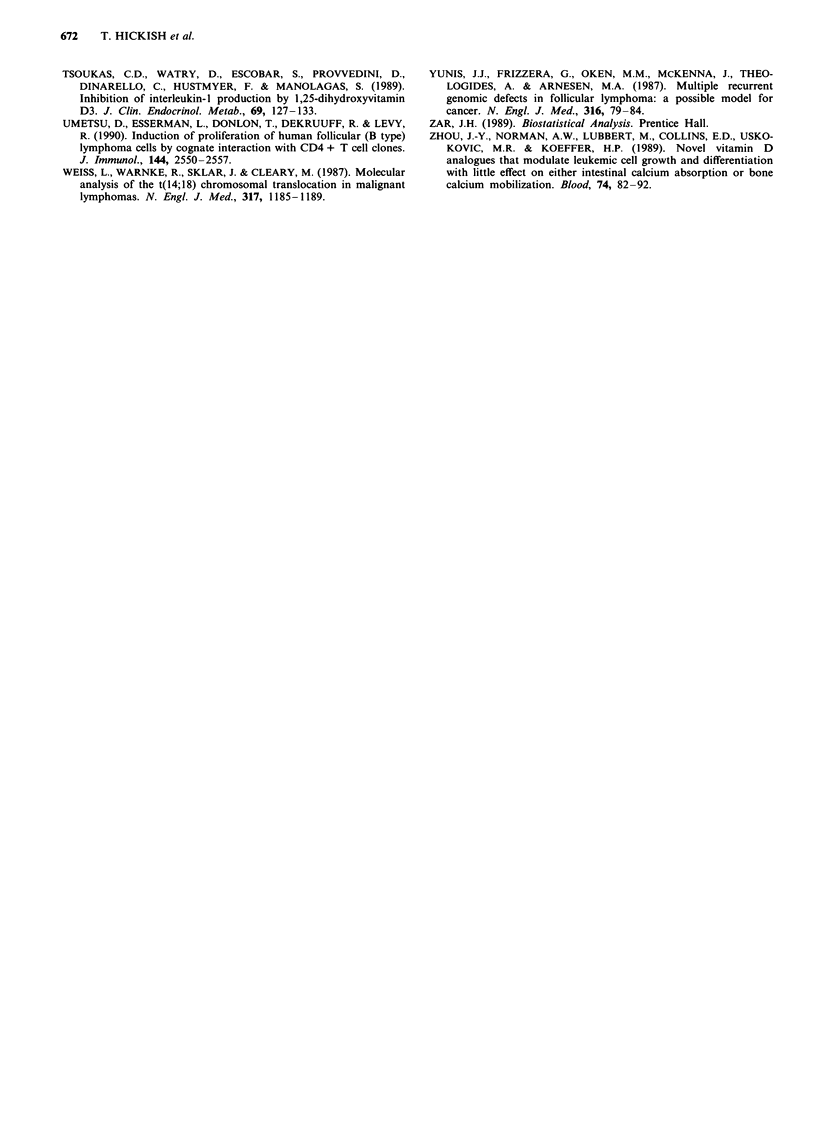# The effect of 1,25-dihydroxyvitamin D3 on lymphoma cell lines and expression of vitamin D receptor in lymphoma.

**DOI:** 10.1038/bjc.1993.406

**Published:** 1993-10

**Authors:** T. Hickish, D. Cunningham, K. Colston, B. C. Millar, J. Sandle, A. G. Mackay, M. Soukop, J. Sloane

**Affiliations:** Lymphoma Unit, Royal Marsden Hospital, Sutton, Surrey, UK.

## Abstract

**Images:**


Br. J. Cancer (1993), 68, 668-672 © Macmillan Press Ltd., 1993

### The effect of 1,25-dihydroxyvitamin $D_3$ on lymphoma cell lines and expression of vitamin D receptor in lymphoma

T. Hickish[1], D. Cunningham[1], K. Colston[4], B.C. Millar[1], J. Sandle[2], A.G. Mackay[4], M. Soukop[5] & J. Sloane[3]

[1]*Lymphoma Unit and Section of Medicine,* [2]*Section of Immunology and* [3]*Department of Histopathology, Institute of Cancer Research, Royal Marsden Hospital, Sutton, Surrey SM2 5PT;* [4]*Department of Clinical Biochemistry, St. Georges Hospital, Medical School, London SW17;* [5]*Department of Medical Oncology, Glasgow Royal Infirmary, Glasgow G4 0SF, UK.*

**Summary** $1,25(OH)_2D_3$ promotes differentiation and has an antiproliferative effect in a variety of cell lines derived from the immunohaematopoetic system. α-Calcidol which is metabolised to $1,25(OH)_2D_3$ has been shown to produce tumour regression in follicular low grade non-Hodgkin's lymphoma (NHL) and the dose limiting toxicity is hypercalcaemia. The cellular action of $1,25(OH)_2D_3$ is mediated by binding to an intracellular protein, the vitamin D receptor (VDR). We have evaluated the activity of $1,25(OH)_2D_3$ and its non-calcaemogenic analogue MC903 in the SU-DHL4 and SU-DUL5 B cell lines which carry the 14;18 translocation characteristic of follicular NHL, and also the expression of the VDR in a range of B cell NHLs. Both agents induced differentiation and had an antiproliferative effect on the SU-DHL4 and SU-DUL5 cell lines. However this occurred at a relatively high concentration ($10^{-7}$ M) which exceeds the physiological concentration of $1,25(OH)_2D_3$ by approximately $10^3-10^4$-fold. Expression of the VDR was low in each cell line and in the low grade lymphoma tumour samples. To account for the observed clinical response to $1\alpha OHD_3$ (α-calcidol) in follicular NHL a network is suggested whereby $1,25(OH)_2D_3$ modulates the activity of $CD4^+T$ cells which have previously been shown to promote follicle centre cell proliferation. Vitamin $D_3$ analogues may enable serum levels to be achieved which produce a direct action on follicular lymphoma cells without disturbing calcium metabolism.

$1,25(OH)_2D_3$ is the active metabolite of vitamin $D_3$ and its action is mediated by an intracellular receptor (VDR) which binds to DNA (Mangelsdorf *et al.*, 1984; Reichel *et al.*, 1989). The presence of the VDR in cells of the immunohaemopoetic system first indicated $1,25(OH)_2D_3$ may have a role in regulating their activity. $1,25(OH)_2D_3$ has been shown *in vitro* to have an antiproliferative effect and promote differentiation in monoblastic and promyelocytic cell lines, to inhibit differentiation in K562 leukaemia cells, to inhibit IL-1 production, to suppress $CD4^+T$ cell proliferation and IL2 production and inhibit immunoglobulin production by B cells (Jordan *et al.*, 1990; Rebel *et al.*, 1992; Tsoukas *et al.*, 1989; Rigby *et al.*, 1987; Moore *et al.*, 1991; Iho *et al.*, 1986; Tsoukas *et al.*, 1984; Bhalla *et al.*, 1989). In a number of cell lines the degree of response to $1,25(OH)_2D_3$ appears to be dependent on the level of ligand binding (Chen *et al.*, 1986) however the precise relationship is unknown. Furthermore $1,25(OH)_2D_3$ has been shown to modulate expression of its own receptor (Lee *et al.*, 1989; Strom *et al.*, 1989). In actively proliferating lymphocytes VDR expression appears to be high and correlates with proliferative status except in B-cells in which expression is low (Kizaki *et al.*, 1991).

B cell lymphomas have been shown to be sensitive to vitamin $D_3$. In a clinical trial, 1 μg of $1\alpha,25OHD_3$ (α-calcidol) daily (metabolised to $1,25(OH)_2D_3$ in the liver) produced an overall response rate of 30% in advanced follicular B cell non-Hodgkin's lymphoma (NHL) (Raina *et al.*, 1991). The clinical importance of this finding is that it indicates vitamin $D_3$ may have a role as a maintenance therapy in the setting of minimal residual disease in follicular NHL. Vitamin $D_3$ analogues which have the antiproliferative effect of $1,25(OH)_2D_3$ but lack its effect on calcium metabolism have been evaluated in a number of cell lines (Norman *et al.*, 1990; Zhou *et al.*, 1989; Binderup & Bramm, 1988; Colston *et al.*, 1992). These could be of clinical value as they may enable higher dosages without calcium toxicity.

The basis of the antilymphoma effect is uncertain. A problem in studying the biology of low grade lymphoma is the lack of suitable *in vitro* and *in vivo* models. Low grade lymphoma cells do not proliferate *in vitro* without adulteration for example by immortalisation with the Epstein Barr virus or culture with $CD4^+$ cells (Umetsu *et al.*, 1990) and such interventions clearly will change the nature of the cells studied. An *in vitro* model which may represent some aspects of follicular lymphoma cell biology are those cell lines which have a t(14;18) chromosomal translocation since this abnormality is found in at least 85% of cases of follicular NHL in association with rearrangement of the bcl2 gene (Yunis *et al.*, 1987; Weiss *et al.*, 1987).

The aim of this study was to investigate the antiproliferative effect and the induction of differentiation of vitamin $D_3$ on t(14;18) lymphoma cell lines and relate this to expression of the VDR in lymphoma tumour biopsy material. $1,25(OH)_2D_3$ and an analogue, MC903 (calcipotriol) which has equivalent VDR binding affinity but 100 fold less effect on $Ca^{2+}$ metabolism were assayed.

### Materials and methods

### *Cells*

The cell lines studied for their response to $1,25(OH)_2D_3$ and MC903 were as follows: SU-DHL4 and SU-DUL5 (both derived from high grade B cell lymphoms): each carries a t(14;18) with an associated rearrangement of the bcl2 gene; in SU-DHL4 rearrangement through the major breakpoint region (Cleary *et al.*, 1986*a*) and SU-DUL5 rearrangement through the minor cluster region (Cleary *et al.*, 1986*b*). U937 is a monoblastic cell line for which both $1,25(OH)_2D_3$ and MC903 have been shown to inhibit proliferation and promote differentiation along the monocytic/macrophage pathway. SU-DHL4 cells were a gift from Dr A. Epstein (UCLA, Los Angeles, CA), SU-DUL5 cells were a gift from Dr M. Cleary (Stanford, CA). Cells were cultured in medium supplemented with 10% foetal bovine serum in humidified atmosphere with 5% $CO_2$.

### *$1,25(OH)_2D_3$ and MC903*

$1,25(OH)_2D_3$ and MC903 (provided by Dr L. Binderup, Leo Laboratories, Denmark) were dissolved in 100% ethanol to

Correspondence: D. Cunningham, Lymphoma Unit, Royal Marsden Hospital and CRC Section of Medicine, Institute of Cancer Research, Sutton, Surrey SM2 5PT, UK.
Received 26 January 1993; and in revised form 18 May 1993.

a stock concentration of $10^{-3}$ M and stored at $-20$°C and protected from light. Dilutions of the stock solutions were made in ethanol and then medium. The maximum concentration of ethanol in culture (0.1%) did not influence cell growth.

*Modulation of cell proliferation and analysis of differentiation*

Cells in log phase growth were seeded at $2 \times 10^5$ cell ml$^{-1}$ and either agent (or vehicle = control) were added at the required concentration. Cells were counted daily using a Coulter counter and assayed for viability with Trypan blue. Each experiment lasted 5 days. On days 0 and 4 cells were examined using a panel of monoclonal antibodies to assay for differentiation. The experiment was performed in duplicate and repeated once.

*1,25(OH)$_2$D$_3$ receptor binding assay*

Cells were harvested by centrifugation and washed twice in ice cold phosphate buffered saline. Cells were then homogenised in KTMED ($KCl_2$ 22.4 g l$^{-1}$, Tris Cl 1.21 g l$^{-1}$, sodium molybate 2.06 g l$^{-1}$, EDTA 0.336 g l$^{-1}$, Dithiothreitol 0.62 g l$^{-1}$ $10^7$ cells ml$^{-1}$ were sonicated and then centrifuged 100,000*g* for 1 h. One hundred and ninety µl of cytosol is then added to 10 µl [$^3$H]-1,25(OH)$_2$D$_3$ ($2.6 \times 10^{-8}$ M) either with or without excess radioinert 1,25(OH)$_2$D$_3$. After 4 h incubation receptor-bound [$^3$]-1,25(OH)$_2$D$_3$ was separated from free [$^3$H]-1,25(OH)$_2$D$_3$ with hydroxylapatite (Colston *et al.*, 1980). Cytosol protein concentration was determined by the method of Bradford (Bradford, 1976). The experiment was performed in duplicate and repeated once.

*Tumour samples and assay for VDR expression*

Tumour biopsy specimens were snap frozen and 5 µm cryostat sections were mounted onto poly-l-lysine coated slides and fixed in 4% formaldehyde and methanol and washed in phosphate buffered saline. (Fixed sections were stored at $-20$°C in glycerol/sucrose storage medium before staining.) Sections were analysed for the presence of the VDR using a Vectastain ABC anti-rat alkaline phosphatase kit with the monoclonal antibody 9A7α (provided by Dr J. Wesley Pike). The alkaline phosphatase substrate contained a levamisole block. Sections were processed in duplicate. The MCF-7 cell line which has a high level of VDR expression served as the positive control.

### Results

*Effect of 1,25(OH)$_2$D$_3$ and MC903 on cell proliferation*

These agents inhibited the proliferation of SU-DHL4 and SU-DUL5 with no effect on cell viability (Figure 1) at $10^{-7}$ M. There was no effect on proliferation at lower concentrations. U937 was inhibited at lower concentrations of each as recorded elsewhere (Binderup & Bramm, 1988). At $10^{-7}$ M there was an approximately 50% reduction of proliferation of SU-DHL4 and SU-DUL5 on day 4.

*Effect of 1,25(OH)$_2$D$_3$ and MC903 on induction of differentiation (Table I)*

SU-DHL4 acquired markers of mature B-cell differentiation (B1, B4 and CD38) whilst SU-DUL5 lost a marker of immaturity (CD14) when cultured with each agent at $10^{-7}$ M.

U937 has been demonstrated to differentiate along the monocyte/macrophage pathway when cultured in the presence of 1,25(OH)$_2$D$_3$ and MC903 (Dodd *et al.*, 1983; Binderup & Bramm, 1988; Bhalla *et al.*, 1989) and this was confirmed in these experiments.

**Figure 1** Growth curves (means ± s.e.m.) for the SU-DHL4 and SU-DUL5 cell lines after culture in the presence of vehicle (control), 1,25(OH)$_2$D$_3$ and MC903 each at a concentration of $10^{-7}$ M. Cell viability was unaffected. There was no effect of either agent at lower concentrations ($10^{-8}$ to $10^{-9}$ M, data not shown).

**Table I** Effect of 1,25(OH)$_2$D$_3$ and MC903 on induction of differentiation

| *Immunocytochemistry profile* | | *VDR level (fmol mg$^{-1}$)* | | |
|---|---|---|---|---|
| *SU-DHL4* | | T = 0 | = 96 h | |
| Controls | No change | 12.0 | 12.3 | NS |
| MC903 | ↑ B1, ↑ B4, ↑ CD38 | 12.0 | 12.3 | NS |
| 1,25(OH)$_2$D$_3$ | ↑ B1, ↑ B4, ↑ CD38 | 12.0 | 9.7 | $P<0.001$ |
| *SU-DUL5* | | | | |
| Controls | No change | 10.4 | 9.9 | NS |
| MC903 | ↓ CD14 | 10.4 | 9.6 | NS |
| 1,25(OH)$_2$D$_3$ | ↓ CD14 | 10.4 | 12.7 | $P<0.05$ |
| *U937* | | | | |
| Controls | No change | 97.5 | 95.7 | NS |
| MC903 | ↑ CD14 | 115.5 | 113.0 | NS |
| 1,25(OH)$_2$D$_3$ | ↑ CD14 | 94.6 | 112.0 | NS |

(1,25(OH)$_2$D$_3$, MC903 at $10^{-7}$ M)

*Effect of 1,25(OH)$_2$D$_3$ and MC903 and VDR expression (competitive binding assay) (Table I)*

Expression of VDR was low in SU-DHL4 and SU-DUL5 both before and after treatment with each agent over 5 days at $10^{-7}$ M in comparison to the U937 cell line. One way analysis of variance was applied to the data. Dunnet's test (Zar, J.H.) demonstrated that incubation with 1,25(OH)$_2$D$_3$ over 96 h produced a reduction in VDR expression in the SU-DHL4 cells ($P<0.001$, 2 tailed test) and an increase in VDR expression in the SU-DUL5 cells ($P<0.05$, 2 tailed). MC903 had no effect on VDR expression in either cell line.

**Figure 2** Immunostaining with 9A7α for the VDR. **a**, The MCF7 cell line – expresses the VDR abundantly. **b**, A lymph node biopsy from a case of follicular mixed small cleaved and large cell lymphoma (working formulation category C). Note weak positive staining and occasional intensely staining cells which in the section shown are macrophages. **c**, A lymph node biopsy from a case of lymphoblastic lymphoma. The tumour cells are strongly positive.

Neither agent significantly altered expression of the VDR at 96 h in the U937 cell line.

### *VDR expression; immunocytochemistry of lymph node biopsy specimens*

Results are summarised in Table II (and see Figure 2). Biopsy samples were analysed for VDR expression from 13 patients with various categories of NHL as defined by the Working Formulation. VDR expression was detectable in 11/13 samples. Only in a case of high grade lymphoblastic-lymphoma was there strong staining comparable to the MCF-7 cell line. In the two negative cases, the macrophages stained positively providing an internal positive control. In all cases macrophages stained strongly positive as did cells in the paracortex which by morphology and location were considered to be T cells.

**Table II** Intensity of staining for the VDR in relation to histological classification according to the working formulation

| *Patient* | *Histology* | *Intensity of staining* |
|---|---|---|
| M.B. | WF: B | Weak +ve |
| L.S. | C | Not detected |
| M.L. | C | Not detected |
| T.F. | C | Weak +ve |
| G.H. | C | Weak +ve |
| K.K. | C | Weak +ve |
| J.P. | G | Weak +ve |
| A.M. | Tranformed F to H | Weak +ve |
| R.M. | G | Moderate +ve |
| J.J. | H | Weak +ve |
| M.G. | Richters | Weak +ve |
| J.L. | J | Strong +ve |
| M.S. | J | Moderate +ve |
| MCF-7 | Breast cancer cell line | Strong +ve |

### Discussion

$1,25(OH)_2D_3$ and its analogue, MC903, had an equivalent antiproliferative effect on the SU-DHL4 and SU-DUL5 cell lines and this was associated with the induction of differentiation. The expression of the VDR was low in both cell lines in accord with other studies (Kizaki *et al.*, 1991) but was slightly altered ($\pm 20\%$) by incubation with $1,25(OH)_2D_3$ and not MC903 over 96 h. The reason for the apparent discrepancy between the two drugs in impact on VDR expression, particularly as they had a similar effect on proliferation, is unclear. The time-course of VDR modulation in response to each drug may differ and hence the response to MC903 may have been missed. The observation that $1,25(OH)_2D_3$ produced a reduction in VDR expression in SU-DHL4 and an increase in SU-DUL5 on day 4 may also reflect different time-course functions of VDR modulation in each cell line.

However these effects occurred at a relatively high concentration ($10^{-7}$ M) of $1,25(OH)_2D_3$ which exceeds the physiological concentration by approximately $10^3-10^4$ fold. Furthermore expression of VDR in the follicular NHL tumour samples was low. These data suggest that the observed clinical response of advanced follicular NHL to $1,25(OH)_2D_3$ may not be due to a direct action of the agent on the lymphoma cells. CD4$^+$T helper cells which recognise alloantigens expressed by follicular lymphoma cells induce the lymphoma cells to proliferate (Umetsu *et al.*, 1990) indicating a possible role for CD4$^+$T cells in promoting follicular NHL. $1,25(OH)_2D_3$ inhibits CD4$^+$T cell proliferation over a concentration range $10^{-8}-10^{-11}$ M and this effect on CD4$^+$T cells appears to be both direct and also indirect through suppression of IL1 production by monocytes (Jordan *et al.*, 1990; Tsoukas *et al.*, 1989; Rigby, 1988; Binderup, 1992). Therefore the antifollicular NHL effect of $1,25(OH)_2D_3$ may be mediated (at least in part) by an inhibitory effect on CD4$^+$T cells. The development of the bcl2 transgenic mouse (McDonnell *et al.*, 1989) provides a novel model of follicular NHL and may enable an *in vivo* analysis of the interplay between B and T cells under the influence of $1,25(OH)_2D_3$ and its analogues. Certainly the t(14;18) cell lines are limited as a model of follicular NHL since they are derived from high grade B cell lymphomas. Nevertheless, if the antiproliferative and differentiation-promoting effect produced by $1,25(OH)_2D_3$ on the SU-DHL4 and SU-DUL5 cell lines extends to lymphoma of follicle centre cell type, then using $1,25(OH)_2D_3$ analogues it may be possible to achieve serum levels that act directly on the centrocytes, in addition to the postulated indirect mechanism of T cell inhibition, without perturbing calcium metabolism.

This work was supported in part by the Cancer Research Campaign.

### References

BHALLA, A., AMENTO, E.P., SCROG, B. & GLIMCHER, L.G. (1984). 1,25-Dihydroxyvitamin D3 inhibits antigen-induced T-cell activation. *J. Immunol.*, **133**, 1748–1752.

BHALLA, A., WILLIAMS, M., LAL, S. & LYDYARD, P. (1989). 1,25-Dihydroxyvitamin D3, but not retinoic acid, induces the differentiation of U937 cells. *Clin. Exp. Immunol.*, **76**, 274–277.

BINDERUP, L. & BRAMM, E. (1988). Effects of a novel vitamin D analogue MC903 on cell proliferation and differentiation *in vitro* and on calcium metabolism *in vivo*. *Biochem. Pharmacol.*, **37**, 889–895.

BINDERUP, L. (1992). Immunological properties of vitamin D analogues and metabolites. *Biochem. Pharmacol.*, **43**, 1885–1892.

BLIFELD, C., PREHN, J.L. & JORDAN, S.C. (1991). Stimulus-specific modulation of TNF and IL-1-beta gene expression in human peripheral blood mononuclear cells and monocytoid cell lines. *Transplantation*, **51**, 498–503.

BRADFORD, M. (1976). A rapid and sensitive method for the quantitation of microgram quantities of protein using the principal of protein dye binding. *Anal. Biochem.*, **72**, 248.

CHEN, T., LI, J., VAN YE, T., CONE, C. & FELDMAN, D. (1986). Hormonal response to 1,25-dihydroxyvitamin D3 in cultured mouse osteoblast-like cells – modulation by changes in receptor level. *J. Cell Physiol.*, **126**, 21–28.

CLEARY, M., GALILI, N. & SKLAR, J. (1986*a*). Detection of a second t(14;18) breakpoint cluster region in human follicular lymphomas. *J. Exp. Med.*, **164**, 315–320.

CLEARY, M., SMITH, S. & SKLAR, J. (1986*b*). Cloning and structural analysis for cDNAs for bcl-2 and a hybrid bcl-2/immunoglobulin transcript resulting from the t(14;18) translocation. *Cell*, **47**, 19–28.

COLSTON, K., CHANDER, S., MACKAY, A. & COOMBES, R.C. (1992). Effects of synthetic vitamin D analogues on breast cancer cell proliferation *in vivo* and *in vitro*. *Biochem. Pharmacol.*, **44**, 693–702.

COLSTON, K., HIRST, M. & FELDMAN, D. (1980). Organ distribution of the cytoplasmic 1,25 dihydroxycholecalciferol receptor in various mouse tissues. *Endocrinology*, **107**, 1916–1922.

DODD, R., COHEN, M., NEWMAN, S. & GRAY, T. (1983). Vitamin D metabolites change the phenotype of monoblastic U937 cells. *Proc. Natl Acad. Sci. USA*, **80**, 7538–7541.

IHO, S., TAKAHASHI, T., KURA, F., SUGIYAMA, H. & HOSHINI, T. (1986). The effect of 1,25-dihydroxyvitamin D3 on *in vitro* immunoglobulin production in human B cells. *J. Immunol.*, **236**, 4427–4430.

JORDAN, S., LEMIRE, J., SAKAI, R., TOYODA, M. & ADAMS, J. (1990). Exogenous interleukin-2 does not reverse the immunoinhibitory effects of 1,25-dihydroxyvitamin D3 on human peripheral blood lymphocyte immunoglobulin production. *Molecular Immunol.*, **27**, 95–100.

KIZAKI, M., NORMAN, A., BISHOP, J., LIN, C.-W., KARMAKER, A. & KOEFFLER, H. (1991). 1,25-Dihydroxyvitamin D3 receptor RNA: expression in hematopoietic cells. *Blood*, **77**, 1238–1247.

LEE, Y., INABA, M., DELUCA, H. & MELLON, W. (1989). Immunological identification of 1,25-dihydroxyvitamin D3 receptors in human promyelocytic cells (HL-60) during homologous regulation. *J. Biol. Chem.*, **264**, 13701–13705.

MANGELSDORF, D., KOEFFLER, H., DONALDSON, C., PIKE, J. & HAUSSLER, M. (1984). 1,25-Dihydroxyvitamin D3-induced differentiation in a human promyelocytic leukemia cell line (HL-60): receptor-mediated maturation to macrophage-like cells. *J. Cell Biol.*, **98**, 391–398.

MCDONNELL, T., DEANE, N., PLATT, F., NUNEZ, G., JAEGER, U., MCKEARN, J. & KORSMEYER, S. (1989). Bcl-2 immunoglobulin transgenic mice demonstrate extended B cell survival and collicular lymphoproliferation. *Cell*, **57**, 79–88.

MOORE, D.C., CARTER, D.L., BHANDEL, A.K. & STUZINSKI, G.P. (1991). Inhibition by 1,25-dihydroxyvitamin D3 of chemically induced erythroid differentiation of K562 cells. *Blood*, **77**, 1452–1461.

NORMAN, A., ZHOU, J., HENRY, H., USKOKOVIC, M. & KOEFFLER, P. (1990). Structure-function studies of analogues of 1a,25-dihydroxyvitamin D3: differential effects on leukemic cell growth, differentiation, and intestinal calcium absorption. *Cancer Res.*, **50**, 6857–6864.

RAINA, V., CUNNINGHAM, D. & SOUKOP, M. (1991). Alfacalcidol is a nontoxic, effective treatment of follicular small-cleaved cell lymphoma. *Br. J. Cancer*, **63**, 463–465.

REBEL, V.I., OSSENKOPPELE, G.J., VAN DE LOOSDRECHT, A., WIJERMANS, P.W., BEELEN, R.H. & LANGENHUIJSEN, M.M. (1992). Monocytic differentiation induction of HL-60 cells by MC903, a novel vitamin D analogue. *Leukemia Res.*, **16**, 443–451.

REICHEL, H., LOEFFLER, H. & NORMAN, A. (1989). The role of the vitamin D endocrine system in health and disease. *N. Engl. J. Med.*, **320**, 980–991.

RIGBY, W., DENOME, S. & FANGER, M. (1987). Regulation of lymphokine production and human T-cell activation by 1,25-dihydroxyvitamin D3. *J. Clin. Invest.*, **79**, 1659–1664.

RIGBY, W.F. (1988). The immunobiology of vitamin D. *Immunol. Today*, **9**, 54–58.

STROM, M., SANDGREN, M., BROWN, T. & DELUCA, H. (1989). 1,25-Dihyroxyvitamin D3 up-regulates the 1,25-dihydroxyvitamin D3 receptor *in vivo*. *Proc. Natl Acad. Sci. USA*, **86**, 9770–9773.

TSOUKAS, C.D., PROVVEDINI, D.M. & MANOLAGAS, S.C. (1984). 1,25 Dihydroxyvitamin D3: a novel immunoregulatory hormone. *Science*, **224**, 1438–1440.

TSOUKAS, C.D., WATRY, D., ESCOBAR, S., PROVVEDINI, D., DINARELLO, C., HUSTMYER, F. & MANOLAGAS, S. (1989). Inhibition of interleukin-1 production by 1,25-dihydroxyvitamin D3. *J. Clin. Endocrinol. Metab.*, **69**, 127–133.

UMETSU, D., ESSERMAN, L., DONLON, T., DEKRUUFF, R. & LEVY, R. (1990). Induction of proliferation of human follicular (B type) lymphoma cells by cognate interaction with CD4 + T cell clones. *J. Immunol.*, **144**, 2550–2557.

WEISS, L., WARNKE, R., SKLAR, J. & CLEARY, M. (1987). Molecular analysis of the t(14;18) chromosomal translocation in malignant lymphomas. *N. Engl. J. Med.*, **317**, 1185–1189.

YUNIS, J.J., FRIZZERA, G., OKEN, M.M., MCKENNA, J., THEOLOGIDES, A. & ARNESEN, M.A. (1987). Multiple recurrent genomic defects in follicular lymphoma: a possible model for cancer. *N. Engl. J. Med.*, **316**, 79–84.

ZAR, J.H. (1989). *Biostatistical Analysis*. Prentice Hall.

ZHOU, J.-Y., NORMAN, A.W., LUBBERT, M., COLLINS, E.D., USKOKOVIC, M.R. & KOEFFER, H.P. (1989). Novel vitamin D analogues that modulate leukemic cell growth and differentiation with little effect on either intestinal calcium absorption or bone calcium mobilization. *Blood*, **74**, 82–92.